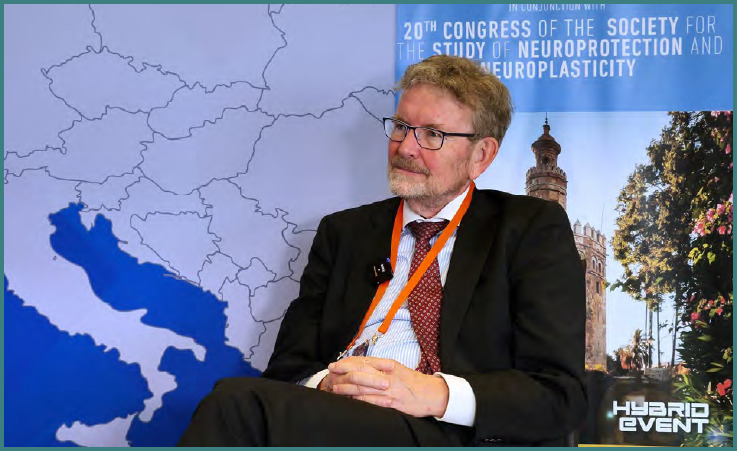# Interview with Prof. Gert Kwakkel - 8^th^ European Congress on Neurorehabilitation in conjunction with the 20^th^ Congress of the Society for the Study of Neuroprotection and Neuroplasticity

**DOI:** 10.25122/jml-2026-1007

**Published:** 2026-04

**Authors:** Stefana-Andrada Dobran, Alexandra Gherman

**Affiliations:** 1 RoNeuro Institute for Neurological Research and Diagnostic, Cluj-Napoca, Romania; 2 Sociology Department, Babes-Bolyai University, Cluj-Napoca, Romania

**Interviewee: Professor Gert Kwakkel**

**Interviewer: Ms. Stefana-Andrada Dobran**

Professor Gert Kwakkel is a leading scientist in stroke neurorehabilitation. At the Amsterdam University Medical Center, he holds the chair of Neurorehabilitation, where his research focuses on the critical link between brain plasticity and motor recovery in the early phase following a stroke. With hundreds of highly cited publications and prestigious honors—including an advanced ERC laureate and the Princess Margaret Memorial Award—he is internationally recognized as a key figure in shaping the field. He also serves as an editor for major journals such as Stroke and Neurorehabilitation and Neural Repair and is a visiting professor in the Department of Physical Therapy and Human Movement Sciences at the Feinberg School of Medicine, Northwestern University in Chicago, USA.

**S.D.: Dear Professor Gert Kwakkel, welcome to the 8^th^ European Congress of Neurorehabilitation (ECNR) in conjunction with the 20^th^ Congress of the Society for the Study of Neuroprotection and Neuroplasticity. The ECNR brings together the scientific and clinical communities. What do you believe is the unique role it plays in bridging the gap between research and daily patient care in neurorehabilitation?**


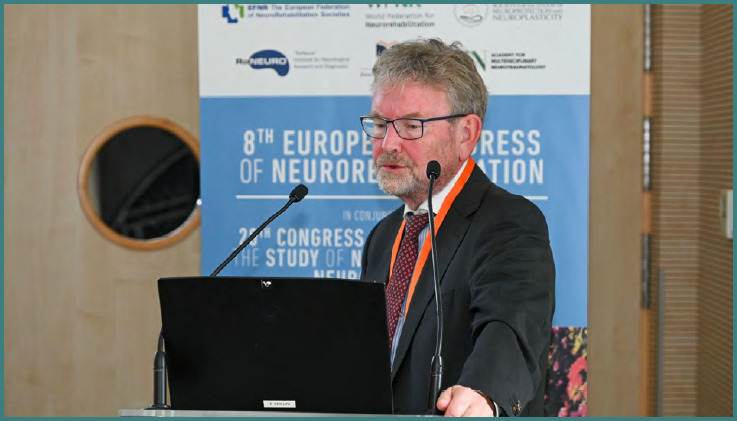


G.K.: I think they are important just to invite people who conduct important research. The role is to improve communication between the different countries within Europe. I also think it's important that the European Federation collaborates with other societies because in Australia, Canada, and the United States, they have the same problems as we do—and they also have societies. So let's bridge the gap by facilitating the activities of this European Federation, making connections with other worldwide initiatives to improve rehabilitation.

**S.D.: Considering your specialty, what future developments do you envision for the complex multidisciplinary field of neurorehabilitation?**

G.K.: It’s a tough question, considering my specialty. I started in the ‘80s as a physical therapist and subsequently studied movement sciences, earned my PhD, and now I’m a professor in this field. But yes, I’m solving a little piece of a very big puzzle.

I think the next step is that we should collaborate. In the field of neuroimaging, for example, there are very big breakthroughs. We have thrombectomy and thrombolysis and other achievements that help us understand what drives stroke recovery—and that’s different from Multiple Sclerosis or Parkinson's disease. There are simply too many pieces and too many diagnoses.

As a movement scientist trying to understand motor plasticity, we need to connect movement sciences with neuroscience. If you make that connection, you will gain a better understanding of what these macroscopic changes in Functional Magnetic Resonance Imaging (fMRI) or electroencephalography (EEG) really represent.

**S.D.: Is there a specific trend or technology that you are excited about from the field?**


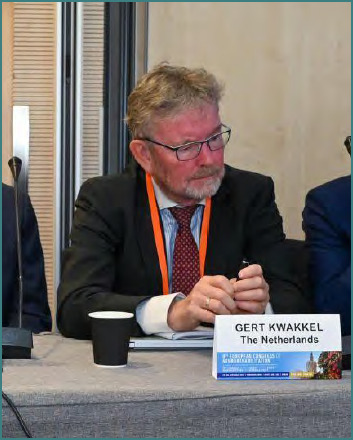


G.K.: There is none, because we are struggling with using technology. Technology is interesting, and you have to distinguish between therapeutic technology—such as robotics or other tools used to improve behavior or cognitive impairments—and on the other side, the monitoring component. I think the most and biggest achievements have been gained on the monitoring side. We are now better at measuring the quality of movement, for example, and what patients are doing outside the laboratory. Having gait labs and similar tools—that’s the biggest achievement so far. Not robotics, not transcranial direct current stimulation (TDCS) or these kinds of things, because we think they can help patients, but the evidence is still very weak.

**S.D.: From your perspective, what is the most challenging future development in neurorehabilitation and how can the European Federation of Neurorehabilitation Societies (EFNR) come closer to this endeavour?**

G.K.: If you consider the development of stroke rehabilitation, which is my particular focus, you need to better understand the pathology and why one patient recovers while another does not. If you don't understand this heterogeneity in recovery patterns—how some patients improve over time, some don't improve, and some improve within days or weeks after a stroke—it is hard to design interventions and understand what an intervention does on top of these natural recovery patterns. So, the first low-hanging fruit is to start by understanding better why our patients recover or do not recover after a stroke.

For this purpose, you need to collaborate with acute neurologists, recognizing that stroke is defined in the first 24 hours. We see this through thrombectomy and the role of good collaterals in the brain, factors that allow some patients to walk again and go home even after thrombectomy. Therefore, to understand why patients recover months and even years after the incident, and how rehabilitation interacts with this process, you need to collaborate with acute neurologists as well as all the disciplines involved after discharge from the stroke unit.

**S.D.: What do you see as the most significant barrier preventing our understanding of neuroplasticity from being effectively translated into reliable paradigms and how can we overcome that?**

G.K.: The brain is very plastic, and we know that. However, interpretation is another matter. We can see that the brain is adapting with new networks in EEG, showing coherence—for example, in musculoskeletal EEG coherence using high-density EEG—and we can follow these patients through repeated measurements to understand what these plastic changes actually mean.

We know there is motor learning, which involves plastic changes, but there are also plastic changes that are independent of motor learning. To truly understand these plastic changes, you need to know exactly what is being learned and how patients are changing on the outside. Therefore, I think there is an opportunity to connect, for instance, movement science with neuroscience and these plastic changes, in order to grasp what these changes are doing in the brain.